# Developing a graduate training program in Synthetic Biology: SynBioCDT

**DOI:** 10.1093/synbio/ysz006

**Published:** 2019-01-30

**Authors:** Idil Cazimoglu, Alexander P S Darlington, Aurelija Grigonyte, Charlotte E G Hoskin, Juntai Liu, Robert Oppenheimer, Jesús A Siller-Farfán, Claire Grierson, Antonis Papachristodoulou

**Affiliations:** 1 Department of Chemistry, University of Oxford, South Parks Road, Oxford, UK; 2 EPSRC and BBSRC Centre for Doctoral Training in Synthetic Biology, Doctoral Training Centre, University of Oxford, South Parks Road, Oxford, UK; 3 School of Engineering, University of Warwick, Library Road, Coventry, UK; 4 School of Life Sciences, University of Warwick, Gibbet Hill Campus, Coventry, UK; 5 School of Biochemistry, University of Bristol, Biomedical Sciences Building, University Walk, Bristol, UK; 6 Department of Physics, University of Oxford, Parks Road, Oxford, UK; 7 Sir William Dunn School of Pathology, University of Oxford, South Parks Road, Oxford, UK; 8 School of Biological Sciences, University of Bristol Bristol Life Sciences Building, 24 Tyndall Ave, Bristol, UK; 9 Department of Engineering Science, University of Oxford, Parks Road, Oxford, UK

**Keywords:** Synthetic Biology, graduate education

## Abstract

This article presents the experience of a team of students and academics in developing a post-graduate training program in the new field of Synthetic Biology. Our Centre for Doctoral Training in Synthetic Biology (SynBioCDT) is an initiative funded by the United Kingdom’s Research Councils of Engineering and Physical Sciences (EPSRC), and Biotechnology and Biological Sciences (BBSRC). SynBioCDT is a collaboration between the Universities of Oxford, Bristol and Warwick, and has been successfully running since 2014, training 78 students in this field. In this work, we discuss the organization of the taught, research and career development training. We also address the challenges faced when offering an interdisciplinary program. The article concludes with future directions to continue the development of the SynBioCDT.

## 1. Introduction

Synthetic Biology is a new field that aims to engineer new or modified biological components and systems (1–4). This subject connects a wide range of computational and experimental topics; typical undergraduate courses cover only some of the disciplines embodied in Synthetic Biology. Post-graduate training of the field’s next industrial and academic leaders requires training in all these aspects, in order to provide them with the knowledge to make advances in this area. These skills can be gained through theoretical and experimental modules, group discussions and presentations, as well as research in multidisciplinary environments. The United Kingdom recognized this need early: in its 2012 Roadmap (5), the country recommended the creation of a ‘skilled, energized and well-funded UK-wide synthetic biology community’.

Here, we describe our experience in developing and running a 4-year doctoral training program in Synthetic Biology, responding to this recommendation. The Centre for Doctoral Training in Synthetic Biology (SynBioCDT) was created in 2014, and is currently funded through the Engineering and Physical Sciences and the Biotechnology and Biological Sciences Research Councils (EPSRC and BBSRC). Both the EPSRC and BBSRC are part of UK Research and Innovation, an organization that supports science and research in the country. The SynBioCDT was designed as a collaboration between the Universities of Oxford, Bristol and Warwick, and offers 6 months of taught training, followed by two 3-month exploratory research projects. The short projects allow students to experience research in two different laboratories, enabling them to make an informed choice on their doctoral project. The following 3 years are aimed at a substantive research project. Our training program is broad and of adequate depth, taking advantage of the strengths of the three institutions across disciplines and bringing together students and supervisors by promoting the sharing of knowledge and facilities.

## 2. SynBioCDT: the training program

**Figure 1.**
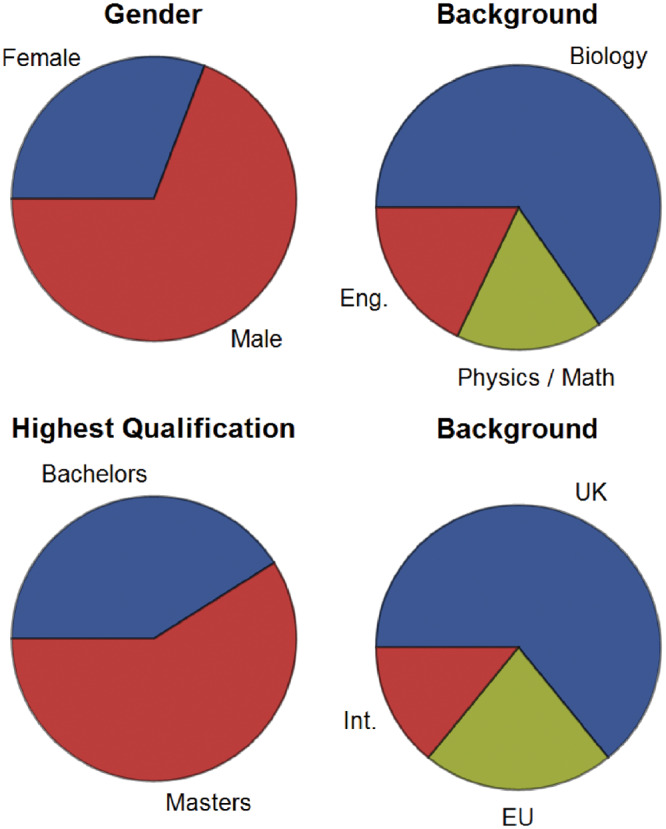
Student demographics. The gender, national and academic background of the SynBioCDT for all intakes 2014–2018. *N *=* *78. Note that in ‘academic background’ subjects are combined into their larger subject areas of biology, physical sciences (including chemical sciences and mathematics), and Engineering (abbreviated to Eng.). Nationality data are broken down by funding status; UK nationals, other European Union (EU) nationals and International students (all others who do not qualify in the previous two groups, abbreviated to Int.).

As an interdisciplinary training program, the CDT attracts and recruits students from across the Natural Sciences, Mathematics and Engineering (Figure 1) all of whom are keen to undertake an interdisciplinary doctorate in Synthetic Biology. Sixty percent of students had a Master’s degree upon starting the program (Figure 1). The students come from around the globe.

**Figure 2.**
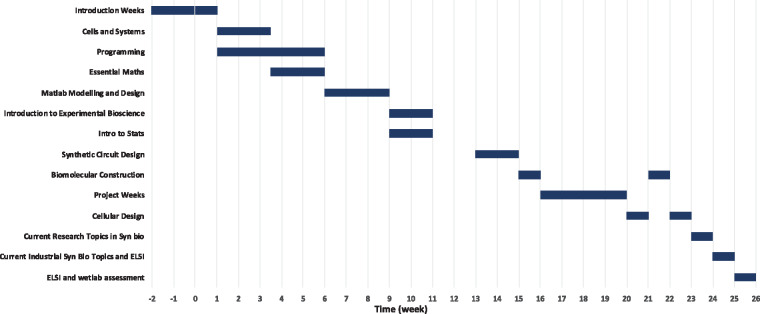
Timeline of SynBioCDT training courses in the first 6 months.

The taught part of the program consists of two terms and is delivered through 14 short modules covering essential and advanced topics relevant to Synthetic Biology (Figure 2). Given the diversity of the students’ background as shown in Figure 1, students initially take introductory courses complementary to their undergraduate degree to ensure all of them have the necessary knowledge for further study. For example, students with biology backgrounds study ‘Essential Mathematics’ and students from the physical sciences and engineering/mathematics take ‘Cells and Systems’ (Table 1). Following this period, students are taught as a single cohort, which fosters a collaborative and interdisciplinary working environment.

**Table 1.** Modules read by students during the initial training period.

Module	Example content	Teaching	Assessment
Cells and systems (2-week introductory course taken by physical sciences/engineering students)	Cell biology Molecular biology (DNA replication, transcription, translation) Signal transduction Metabolism Molecular genetics	Lectures Group discussion and projects	Group project and presentation
Essential mathematics (2-week introductory course taken by biologists/biochemists)	Matrix algebra Differentiation and integration Systems of ODEs Complex numbers Eigenvalues and vectors Sequences and series Introduction to (i) vector calculus and (ii) Partial Differential Equations (PDEs) Statistics	Lectures Problem exercises	Exercises
Programming (2 weeks)	Computer architecture Typed variables Loops Arrays Functions and subroutines Data structures File I/O	Theory lectures Problems	Report based on a 3-day programming project
Introduction to systems and synthetic biology (1 week)	Static modeling of metabolism Dynamic modeling of gene expression Dynamic modeling of signaling cascades	Lectures Pen and paper exercises	Assessed through group presentation
MATLAB (2 weeks for core material, 1 week advanced material and group project)		Lectures Problems	Group project and presentation
Synthetic circuit design (2 weeks)	Logic gates Part standardization Chassis engineering CAD tools (e.g. Clotho, GEC compiles) Promoter engineering RNA secondary structure engineering Ribosome Binding Site strength engineering RNA circuits Protein engineering	Lectures Discussion	Presentation of a simple circuit design including discussion of implementation steps
Cellular design I (1 week)	Metabolic Networks, Inc. discovery of gene clusters Rational design of metabolic pathways Plant synthetic biology Genome engineering	Lectures	Presentation of a paper of the student’s choice
Cellular design II (1 week)	Synchronization/Quorum sensing Biofilms Agent-based modeling Modeling of microbial communities	Lectures In-class exercises	
Biomolecular construction I (1 week)	Natural production, synthesis etc. of polypeptides and nucleic acids Protein engineering Protocells Membrane proteins Applications of modified nucleic acids	Lectures Discussions	Presentation of a topic of the student’s choice
Biomolecular construction II (1 week)	Biopolymer production DNA/RNA nanostructures De novo protein design Natural motors/synthetic mimics	Lectures Discussions	
Current research topics in Synthetic Biology (1 week)	Systems and control theory Retroactivity and scalability Noise and stochasticity Mammalian synthetic biology Impact of evolution	Lectures Mathematical exercises	Submission of exercises
Current industrial topics (1 week)	Mature applications Prospects/challenges (e.g. microbial communities, mammalian cells and plants)	Lectures Lectures from industrial collaborators Discussions	–
Advanced experimental techniques (2 weeks)	Specialist equipment, e.g. microfluidics, robotics	Lectures Practical sessions	
ELSA (1 week)	Ethics Social justice Dual use and biosecurity Public understanding of synthetic biology The role of the social scientist Considerations of futures and expectations Values	Guest lectures by specialists Discussions of set texts	3000 word essay on a topic of the student’s choice

### 2.1 Training in computational and mathematical areas

Students are introduced to programming in C and Python through a 2-week intensive software carpentry course which is assessed through a short project. Afterwards, MATLAB and mathematical modeling are introduced through demonstrations and exercises. This consists of a series of lectures covering a range of modeling frameworks for metabolism, gene expression and signaling pathways, which are implemented in the MATLAB suite. Progress is assessed through a group project where students are tasked to solve a simple biological question or create a simple toolbox using MATLAB. Of note, groups must be interdisciplinary, namely a mixture of experimentalists and theoreticians. Depending on the specific background of the student, the first term finishes with either a 2-week module on Experimental Bioscience or on Data Analysis and Statistics.

The second term of the training period focuses on key Synthetic Biology research taught by academic and industry experts in the field, as outlined in Table 1. These advanced modules are taught as a single cohort, and combine mathematical and computational elements as required. Specific topics include agent-based models for probing bacterial communities, control theory in the context of genetic feedback devices and stochastic modeling methods applied to gene expression noise. Assessment of these modules is formative: students carry out a short computational project or study a paper from the literature, on which they lead a discussion.

### 2.2 Training in biological and biophysical areas

All members of the cohort also attend advanced modules on biological and biophysical topics. This enables them to understand concepts related to synthetic circuits, DNA/RNA nanostructures, as well as metabolic and protein engineering. The program also features instruction related to gene cluster discovery and genetic engineering. Importantly, students are introduced to a number of software suites routinely used by synthetic biologists, including Eugene (6), Visual Genetic Engineering of Cells (GEC) (7), Cello Computer Aided Design (CAD) (8) and Snapgene (www.snapgene.com). Recognizing the importance of synergies between academia and industry, the SynBioCDT also devotes a module to discussing the current state of this discipline in a commercial setting. Agriculture, biofuel production, bioremediation and other activities are dissected from different frameworks, ultimately enabling students to reason the strengths, challenges and novel applications of biological engineering.

Moreover, students travel again to Bristol and Warwick to visit—respectively—the BrisSynBio (9) and the Warwick Integrative Synthetic Biology centers (10). This serves two functions. First, those interested in pursuing their PhD at the Universities of Bristol and Warwick visit prospective groups and maintain conversations with Principal Investigators (PIs). Second, the cohort has the opportunity to interact with staff that run facilities in the fields of microscopy, Nuclear Magnetic Resonance (NMR), X-ray crystallography and microfluidics. Where practicable, lectures are complemented with hands-on sessions.

### 2.3 Training in experimental Synthetic Biology

One of the features that distinguishes SynBioCDT is the inclusion of formal training in an array of experimental techniques. Even before students carry out their project rotations, they have the opportunity to construct and characterize genetic circuits as well as DNA nanostructures. The reader should note the wetlab training is open-ended: in the beginning of this module, students devise a hypothesis related to a standard genetic circuit. Afterwards, they have the opportunity to engineer the key components (promoters, ribosome binding sites and degradation tags) to test their predictions. This module also includes a brief introduction to biomimetic construction; here the cohort designs, constructs and verifies—using atomic force microscopy—a DNA nanostructure. Depending on their specific background, this enables students to learn or reinforce concepts related to molecular cloning, microscopy, quantitative PCR and spectrophotometry. This training is particularly relevant for students with little or no experience in a wetlab environment, as it gives them confidence to pursue an experimental project should they wish to do so.

### 2.4 ELSA training

Students are introduced to the core concepts and approaches adopted in the ethical, legal and social aspects (ELSA) of Synthetic Biology (11) through a series of lectures and discussion groups. This encourages them to critically reflect on their own and others’ assumptions about Synthetic Biology. Upon completion of the module, students write a 3000-word essay and literature review on an emerging topic of their own choosing in ELSA and Responsible Research and Innovation (RRI) (11, 12). In past years, essays produced by the cohort covered a diverse range of topics, from the ethics of human gene modification to the implications of dual-use (commercial and military) techniques.

To encourage our students to consider the impact of their own project and to highlight that science is not isolated from the wider social context, funds are available to support outreach activities both locally and at UK-wide science festivals.

### 2.5 Industrial engagement

The SynBioCDT has developed collaborations with over 10 industrial partners and, to date, more than 10 students have done projects directly related to industry. Several laboratories hosting SynBioCDT doctoral students have spin-out companies where students benefit from the entrepreneurial environment. Some scholarships follow an iCASE format, where partners provide most of the funding and propose a project with an industrial scope (13). During the initial CDT training year, students explore mature applications of Synthetic Biology through a dedicated ‘Current Industrial Synthetic Biology’ module. This covers the potential applications and challenges to commercialization in emerging areas such as engineering of microbial communities.

In the later years of the program, a number of CDT training events continue to take place. During these events, speakers from industry are frequently invited to address and meet with students. Students value these opportunities to learn more about the state of the art in the bioprocessing and biotechnological industries and the potential career paths available to them. In addition to this formal training, the CDT constituent Universities are partners of the UK’s synthetic biology accelerator SynBiCITE (14), and numerous students have taken advantage of courses provided there.

## 3. Research training (exploratory projects)

Rotation projects embrace the interdisciplinary nature of Synthetic Biology. Students are encouraged to take full advantage of their strengths, and the variety of projects offered by the three institutions. The exploratory projects not only provide students with an opportunity for learning new skills but also encourage them to establish collaborations between multiple institutions and prepare them for undertaking their substantive doctoral project.

**Figure 3.**
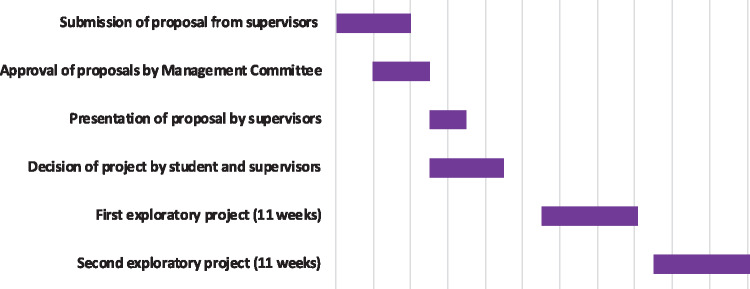
Timeline of SynBioCDT research training.

Research training is comprised of two exploratory projects offered by PIs across the three institutions (see Figure 3). Projects devised by PIs are presented to students who then choose two of them, effectively establishing the groundwork for their doctorate. Since the SynBioCDT has multiple partners in the UK as well as abroad, the cohort has opportunities to establish international collaborations during their studies. The outcome of each exploratory project is presented in a short journal publication-style report and assessed by the supervisor and a second assessor. Both projects often result in significant contributions (see Section 5). After the research training, students make a fully informed decision on the direction of their dissertation for the next 3 years.

## 4. Continuous and career development training (years 2–4)

The SynBioCDT continues to provide students with support and training after they have started their doctorate research. One aspect of this continuous training, beyond a small taught component, is to assist students with their academic careers through workshops run on poster presentation skills, as well as advice on the process of publishing papers. Courses are run alongside these workshops covering other aspects related to scientific divulgation to key stakeholders (policy makers, industry and the public).

Recently, the continuous training program was enhanced with a 2-year ‘Impact beyond academia’ module. Students are introduced to three topics in their first year: communication skills (teaching), public engagement and enterprise. In their second year, cohort members choose which topic most appeals to them and the CDT provides the appropriate training and opportunities to carry out activities within this topic. Following the completion of the topic assignment, students are encouraged to reflect on their experiences and learn from it in order to assist with future endeavors beyond academia.

A unique feature of the program is the requirement for students in their first year of research to organize an event at their home institution. These events not only provide the opportunity to discuss research with students from other institutions or years but allow inter-cohort cohesiveness to develop despite the CDT being multi-institutional. Students are given the freedom to choose what the day involves; previous events have ranged from academic talks to a media training workshop. This provides students with the experience of organizing an academic event and the opportunity to network within the synthetic biology community. The main event of the year is the annual Synthetic Biology CDT Summer School which all cohorts are encouraged to attend. This is a student-led 3-day conference where international speakers from academia, industry and science policy are invited to talk about their work alongside the students. There is also a public engagement aspect of the summer school: 1 year this involved workshops on digital engagement through online writing as well as an opportunity to get involved with University open days and the National iGEM meetup. Other engagement activities included visiting local schools and a public debate held during the Oxford Science Festival. There are also social aspects of the summer school encouraging students to get to know one another as the program encourages collaborative learning.

Through this continuous training, students are provided with the tools to develop a wide range of transferable skills alongside their studies creating well-rounded graduate students who can become the next generation of industrial and academic leaders within the field.

## 5. Student feedback

**Figure 4.**
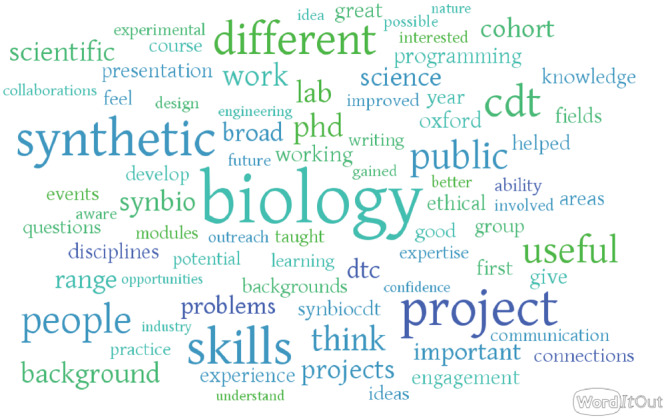
Key words from SynBioCDT student feedback.

It is imperative that a new program actively seeks and incorporates feedback from students. This tests whether the goals of the program are being met, and also helps quickly resolve unanticipated issues and ensures that the experiences of a cohort of students improve the experience of the following cohort. Importantly, the issues students raise span from administration to academic course structure, so to cover everything it is necessary to get student feedback over a variety of time-scales and formats. In the SynBioCDT, student feedback was sought through at least four means: (i) having informal conversations throughout the program, (ii) electing student representatives from the cohort, (iii) online student surveys at the end of every module and (iv) annual surveys. Two recurring themes from SynBioCDT student feedback emphasize ‘confidence’ and ‘connections’ (see Figure 4).

While a 2-week module in a programming language is insufficient to become an expert in programming, the most valuable contribution appears to be increasing student’s confidence. As the core concepts and nomenclature in the area are explained in detail by a lecturer or demonstrator, the barriers for students to continue developing the skill are greatly reduced. Later during their doctoral research, when a student discovers that they need this skill, they know where to look, how to start and what questions to ask for help. Students report that the training has helped them transition between fields, enabling mathematicians to become experimentalists and biochemists to code. This is enhanced by the synergistic cooperation between students during assignments. Similarly, as students are hosted between three different research institutions, some have helped forge and maintain lasting academic and industrial collaborations.

Over the years students have drawn attention to the following issues: (i) due to the various backgrounds of the students for any given course there are beginners and experts receiving the same content, (ii) due to the number and diversity of lecturers in the program, not all may be familiar with content that was previously covered, leading to some lectures assuming different levels of background knowledge, (iii) formative assessment is a new concept for most students, who expect assignments to be formally assessed. All three points have been raised and addressed every year by explicitly stating that (i) lecturers should ensure that the content and pen and paper/computer exercises increases in difficulty so that every student finds some questions challenging and that all students have achieved the required level of understanding by the end of each day, using help from demonstrators as needed, so that they can benefit from the rest of the course, (ii) lecturers should consider previous lecture notes and be flexible when delivering the lecture content ensuring there is engagement by all students (iii) students should strive to become self-aware and autonomous in their own learning process—this is ultimately the skill they will need during their doctorate.

Regular feedback from students has helped to improve the SynBioCDT program over time. For example, in 2016 our students reported that they did not have enough time between lectures to consolidate their knowledge with further reading and research, nor to prepare their best work for assignments. It was also challenging for students to organize meetings with potential supervisors and collaborators when they had full-time lectures with different week-to-week schedules. In response, students now have Wednesday afternoons free during all taught modules, enabling them to use this time effectively as they see fit. Similar changes to module timetabling have been made after students suggested to better align lecture-based modules with their respective laboratory training. A further example of program improvement has been communicating the resources and support available for mental health issues (a new session for first year students has been created, while student handbooks and the website have been updated). These changes were the direct result of a survey conducted by a student representative.

The experiences of students are a valuable resource for refining the future development of the SynBioCDT.

## 6. Future developments and conclusions

To fulfill the needs of the rapidly accelerating field of synthetic biology, our CDT has been continuously evolving to ensure the most realistic, student orientated preparation. Industrial partners from a variety of sectors have been included as collaborators to make the training as relevant to the current opportunities and technologies as possible. Going forward, our CDT aims to reshape the training to encourage student’s further understanding of the key challenges associated with start-up, scale-up and process development. The future CDT training will also support creativity and opportunities for science and innovation that are in the public interest by including an enhanced responsible research innovation component. Our CDT provides its students with a rich understanding of the interdisciplinary nature of synthetic biology and fosters a wide range of skills allowing students to address multiple challenges in this biotechnology sector.
